# Exploring the use of cannabis as a substitute for prescription drugs in a convenience sample

**DOI:** 10.1186/s12954-021-00520-5

**Published:** 2021-07-10

**Authors:** Sinikka L. Kvamme, Michael M. Pedersen, Kristine Rømer Thomsen, Birgitte Thylstrup

**Affiliations:** 1 Center for Alcohol and Drug Research, Aarhus BSS, Aarhus University, Building 1322. Bartholins Allé 10, 8000 Aarhus C, Denmark; 2 Center for Alcohol and Drug Research, Aarhus BSS, Aarhus University, Artillerivej 90, 2. Floor, 2300 Copenhagen S, Denmark

**Keywords:** Substitution, Medical cannabis, Opioid use, Prescription drugs, Cannabidiol

## Abstract

**Background:**

The use of cannabis as medicine (CaM) both prescribed and non-prescribed has increased markedly in the last decade, mirrored in a global shift in cannabis policy towards a more permissive stance. There is some evidence that cannabis functions as a substitute for prescription drugs, particularly opioids; however, more knowledge is needed on the motives of substitution users, their patterns of use, and perceived effects of substitution use.

**Aims:**

To explore who substitutes prescription drugs with cannabis, the type of prescription drugs substituted and the type of cannabis used, and the impact that substitution with cannabis has on prescription drug use as well as the motives for substitution in terms of experienced effects and side effects.

**Methods:**

A self-selected convenience sample was recruited through social media, public media, and patient organizations to take part in an anonymous online survey. Inclusion criteria were 18 years or older and use of cannabis (prescribed or non-prescribed) with a medical purpose.

**Results:**

The final sample included 2.841 respondents of which the majority (91%) used non-prescribed cannabis, and more than half (54.6%) had used CaM with the purpose of replacing a prescribed drug. Compared to non-substitution users, substitution users were more likely to be women and to use CaM in the treatment of chronic pain and other somatic conditions. Pain medication (67.2%), antidepressants (24.5%), and arthritis medication (20.7%) were the most common types of drugs replaced with CaM. Among substitution users, 38.1% reported termination of prescription drug use, and 45.9% a substantial decrease in prescription drug use. The most frequent type of cannabis used as a substitute was CBD-oil (65.2%), followed by ‘hash, pot or skunk’ (36.6%). More than half (65.8%) found CaM much more effective compared to prescription drugs, and 85.5% that the side effects associated with prescription drug use were much worse compared to use of CaM.

**Conclusion:**

CaM is frequently used as a substitute for prescription drugs, particularly opioids. More research is needed on the long-term consequences of use of CaM, including the impact from low and high THC cannabis products on specific somatic and mental health conditions.

## Background

In recent years, there has been a global shift in perspectives on the utility of cannabis. While cannabis has predominantly been associated with recreational and/or problematic use, the plant or its components, *cannabinoids*, are increasingly regarded as a viable treatment option for medical conditions, such as chronic pain, spasticity, nausea, and epilepsy [1], and as a potential treatment of other conditions [2–4].

The medicalization of cannabis is in large part driven by the discovery of the endocannabinoid system in the late eighties, pharmaceutical interests in cannabinoids, and a growth in user demand for access to *medical cannabis* (cannabis prescribed by a doctor) [5, 6]. This development is mirrored in the rapidly shifting policy on the adoption of medical cannabis laws in more than 30 states in the USA [7], Australia [8], Canada [9] and several European countries [10], including Denmark [11]. However, the introduction of medical cannabis into medicine is controversial and highly debated. While critics caution use of medical cannabis due to the limited high-quality evidence [12, 13], proponents argue that medical cannabis constitutes an important harm reduction strategy and may function as a qualified substitute for prescription drugs, particularly opioids [14, 15] in the context of the opioid-epidemic in the USA [16] and Canada [17].

In drug research, the term “substitution” is conventionally associated with the use of opioid antagonists, such as methadone, in the treatment of opioid addiction [18, 19]. The concept of substitution has its origins in behavioral economics and involves the assessment of the interaction of multiple concurrent commodities [20]. According to this concept, a substance is regarded as a *substitute* if it acts as a replacement, or leads to reduced use of another substance (e.g., treating heroin addiction with buprenorphine) [21]. Conversely, two substances may be *complimentary* when the increased consumption of one substance enhances the consumption of the other (e.g., tobacco smoking is often linked to excessive use of alcohol) [22]. Lastly, if there is no interaction on consumption patterns between substances, they are *independent* (e.g., it has been found that a change in price of alcohol has no impact on consumption of ecstasy [23]).

### Substitution of cannabis for prescription drugs

Emerging research indicates that the increased use of CaM (cannabis as medicine) has had a substitution effect on prescription drug use. In several cross-sectional surveys conducted in the USA and Canada users of CaM report substituting cannabis for prescription drugs, of which opioid, anti-depressive, and anxiolytic drugs are the most prevalent [24–29]. In fact, substitution of prescription drugs is the most common motive among users of medical cannabis, surpassing substitution rates for alcohol and illicit drugs [24, 25, 30, 31]. Moreover, state medical cannabis laws in the USA have been associated with a sizeable reduction in prescription drugs [32], particularly opioid prescriptions [33–35], and with fewer prescription opioid-related hospitalizations, lower rates of opioid overdoses, and decreases in opioid-related healthcare costs [36]. A potential substitution effect of CaM on prescription drug use in a European context is much less explored, and findings from the USA in particular, may not be applicable, due to the considerable variations in health care systems [37, 38] and laws on prescription drugs and cannabis. However, a recent Italian study found that an unintended legalization of cannabis products with less than 0.6% *Δ-9-tetrahydrocannabinol* (THC; main psychoactive component of cannabis) between December 2016 and May 2019 [39] was associated with a considerable decrease in pharmacy sales of anxiolytics, sedatives and anti-psychotics, and a moderate decrease in the sale of opioids, anti-depressants, anti-epileptics, and migraine medication [40].

When evaluating the harm reduction potential of substituting prescription drugs with CaM, it is relevant to consider the subtype of cannabis used as a substitute. Emerging research shows that potential harms related to consumption of cannabis depend on the composition of active components (cannabinoids) in the cannabis used, as cannabis products with high levels of THC are more harmful in terms of negative impact on cognitive function, anxiety- and psychotic symptoms, and addiction, compared to low THC-products [41–45]. Moreover, other studies indicate that *Cannabidiol* (CBD; main non-psychoactive component of cannabis) has anxiolytic and antipsychotic effects [43–45], and therapeutic effects on addiction, including opioid, stimulant, and cannabis addiction [46, 47]. Further, some evidence suggests that CBD may protect against some of the harmful effects of THC [44, 48]. Taken together, these findings highlight the relevance of the subtype of CaM used in an evaluation of potential harm reduction related to the substitution effect of CaM.

### Medical cannabis as the “lesser of two evils?”

In light of recent research findings on the substitution effect of CaM on opioid use, CaM has been suggested as a valuable intervention strategy in combatting the ongoing opioid epidemic in the USA and Canada [14–17, 49]. The most frequent rationales behind this suggestion are the substantial evidence that cannabis is effective in treating chronic pain in adults [1], the prevalent use of medical cannabis in pain management [50], and the fact that, unlike opioids, cannabis has no reported deaths due to overdose [51], as acute effects of cannabis do not depress respiratory function [52, 53]. The non-psychoactive component of cannabis, CBD, has been of particular interest as a target for opioid use disorder [16, 54], as CBD has a discrete modulatory effect on the endocannabinoid system compared to the direct action of THC, giving CBD a broader therapeutic range [16]. Also, there may be an opioid-sparing effect of cannabis, as pre-clinical studies show that co-administration of cannabinoids with opioids enables a reduction in opioid dose without loss of analgesic efficacy [55]. On the basis of growing indications that cannabis may be a viable tool in targeting the adverse effects of opioid use, some US states have recently modified their medical cannabis laws, allowing patients to substitute their prescribed opioids with medical cannabis [56]. What is interesting about this development is that the medical value of cannabis appears to be based not only on what cannabis *is,* in terms of the available evidence on its efficacy, but also on what cannabis *is not,* when compared to opioids [14, 15, 17]. This perception has also been described in a qualitative study of physicians in Israel, where cannabis was presented as a justified treatment option and as a “the lesser of two evils” by emphasizing problems in standard medications [57]. However, opponents of this position argue that the current evidence for the use of medical cannabis is weak, that more clinical trials are needed [58, 59], and that other efficacious medications for the treatment of problematic opioid use are under prescribed [60]. Furthermore, some evidence suggests medical cannabis may not function as a long-term substitute for opioids [61], and cannabis use is associated with increased risk of problematic opioid use [62, 63]. Still, despite the recommendations to wait for more valid evidence on the long-term effects of substituting prescription drugs with CaM, several studies indicate that many of the current users of CaM are using cannabis as a substitute for prescription drugs, particularly opioids, and report fewer side effects and better symptom management as their motive for substitution [28, 64].

### Cannabis as a substitute for prescription drugs in a Danish context

In Denmark, a four year Medical Cannabis Pilot Program (MCPP) was initiated in 2018 with the aim of providing a safe and legal framework for prescribed whole plant cannabis to patients with multiple sclerosis, spinal cord injury, chronic pain, or chemotherapy-related nausea, and vomiting [65]. Medical cannabis is not formally categorized in the MCPP as a potential substitute for any prescribed drug, and the Danish Medicines Agency recommends that only patients who have failed to respond to conventionally approved drugs are prescribed medical cannabis [65]. Thus, in Denmark, medical cannabis is not officially regarded as a tool to combat misuse of prescription drugs, but instead as a last resort. Despite the changes in policy, there are indications of a large use of *medicinal* (non-prescribed) cannabis occurring outside the legal framework of the MCPP [11]. In the first survey in Denmark on the use of CaM, we recently found that the vast majority of respondents (90.9%) reported use of CaM without a doctor’s prescription [11]. Moreover, we found that most users of CaM reported limited recreational experience (63.9%) and a preference for low potency CBD-oil (65%) [11]. Of note, there are several other indications that cannabis oil has become more prevalent in Denmark in recent years, including a growth in web-shops selling low-potency cannabis oil [66], an increase in selling of cannabis oil at the illegal open drug scene Christiania [67], and an increase in police confiscations of cannabis oil [68]. All use of cannabis outside the MCPP is illegal, both sale and possession, except from cannabis products containing less than 0.2% THC. Such products are illegal to sell if they are considered to be medicine by the Danish Medicines Agency, but they are not illegal to possess [69].

The MCPP has set the legal parameters for the utility of CaM in Denmark, but we lack knowledge on the parameters users of CaM set in practice; do they substitute prescription drugs with CaM, and do they perceive CaM to be the lesser of two evils in terms of experienced effects and side effects? Examining the use of CaM as a substitute for prescription opioids is of particular public health interest, in the context of the increased use of prescription opioids in the Danish health care system, which has undergone recent media scrutiny in Denmark [70]. As a response, the Danish Medicines Agency started an investigation, which showed that use of the “weaker opioid” Tramadol was driving a substantial part of the rise in opioid use in Denmark [70].

Taken together, we lack knowledge about whether the emerging trend of using CaM in Denmark is related to a motive of replacing prescription drugs. Previous qualitative research found that using cannabis as a substitute for prescription drugs was a motive among Danish growers of cannabis for medicinal purposes, particularly opioids [71]. However, more knowledge is needed on user perspectives on the utility of cannabis as a substitute for prescription drugs, because user perspectives can unveil aspects of illegal drug use unseen by society at large [72]. Also, there is a need for exploring the type of cannabis used as a substitute, as the potential negative health effects of cannabis use varies according to the subtype of cannabis used [73]. Finally, the vast majority of studies have been conducted in the USA and Canada, and we lack studies examining potential use of CaM as substitution for prescription drug use in a European context.

## Methods

### Aims

The aim of the present study was to examine to what extent CaM is used as a substitute for prescription drugs in a convenience sample of Danish users of CaM, and to characterize the substitution users. Furthermore, the study aimed to examine what type of prescription drugs are substituted with CaM, what types of cannabis are used as substitution, and the perceived impact on prescription drug use. Finally, we aimed to compare perceived effects and side effects of CaM compared to prescription drugs used*.*

### Study design

The study was based on data from the first survey on use of CaM conducted in Denmark (for more details, see [11]). A novel survey was developed, inspired by previous studies on users of CaM [27, 74–79]. The full survey consisted of 44 structured questions and 19 possible follow-up questions answered in a Yes/No format, multiple-choice response, or rating scales. The survey took about 15 min to complete, and was available in Danish only. Data were collected through SurveyXact. IP addresses of the respondents were not saved or available to the researchers, as the respondent’s anonymity was considered a greater issue than the possibility of repeated participation [80]. We have previously reported on the motives for use and patterns of use from 3.021 respondents from this survey [11].

### Sampling and recruitment

The survey was made available online to a self-selected convenience sample of users of CaM from July 14th 2018 to November 1st 2018. Inclusion criteria were being 18 years or older and being a user of CaM, either prescribed by a doctor or non-prescribed with a self-perceived medical purpose. Users of CaM who had stopped using cannabis were included in the sample and characterized as former users of CaM. Participants were recruited via online material, flyers, and posters with information about the survey, and a survey link and QR code directing to the survey. The material was disseminated on social media, through patient organizations, in doctors’ offices and hospitals, at the illegal open drug market in Denmark (Christiania), the first Cannabis Expo in Denmark, and via headshops selling cannabis-related items. Furthermore, the survey was made available through Smokeboddy (an app where users monitor potential police presence at Christiania) and was covered by the national media (The Danish Broadcasting Corporation).

### Measures

The full survey included several key domains; sociodemographics, motivation for use, patterns of use, and evaluation of perceived efficacy and adverse effects. In the present study, the following domains were used for the data analyses (see description of the remaining measures in [11]).

#### Current and former users of CaM

Respondents were asked an introductory question: *Do you use CaM?,* and could answer either Yes/No or “I have used CaM, but I stopped”. Respondents who answered “Yes” were defined as current users, while those who indicated having past experience with the use of CaM were defined as former users, and those that answered “No” were not included in the study sample.

#### Users who used CaM as a substitute for prescribed drugs

Respondents were asked if they had experience with using prescription drugs to treat somatic or mental health condition(s). Those who responded positive were asked a follow-up question on whether they had ever used CaM with the purpose of replacing a prescribed drug. Those who answered affirmatively were categorized as “substitution users”, while all other respondents were categorized as “non-substitution users.”

#### Type of drug substituted

Respondents were asked which prescription drugs they had replaced with cannabis and were provided with six response categories: Pain-medication, Anti-depressants, Anti-psychotic, Anti-epileptic, Arthritis-medication, and an “Other” category, and could choose more than one category. Each category was followed with an open-ended question, where respondents were encouraged to write the brand of the prescribed drug.

#### Impact of CaM on prescription drug use

Respondents were asked to evaluate how their use of CaM affected their use of prescription drugs on a 6-point scale from ‘use of prescription drugs has increased substantially’ (1) to ‘use of prescription drugs has decreased substantially’ (5), including an option to indicate cessation of prescription drug use; ‘I have stopped using prescription drugs’ (6).

#### Effects and side effects

Respondents were asked to evaluate the experienced effects and side effects of CaM compared to the substituted prescription drugs on a 5-point Likert scale from ‘CaM is much more effective than prescription drugs’ (1) to ‘prescription drugs are much more effective than CaM’ (5).

### Data analysis

Data analysis was conducted using Stata SE/15 [81] and Nvivo 12 [82]. Figures were produced in Excel 2016 and Word 2016. Descriptive statistics were used to describe sample characteristics, conditions treated, and patterns of use. A histogram was used to assess normality in the variable for a number of conditions. As the variables were not normally distributed, the Mann–Whitney U test [83] was used to assess differences in means between substitution users and non-substitution users. We coded a dummy variable, distinguishing between substitution users and non-substitution users, with substitution users coded as 1 (both former and current users of CaM were include). This variable was used as the dependent variable in the logistic regression analysis. Odds ratios (ORs) from multiple logistic regression, controlled for age, gender, and current employment, were used to estimate the strength of association between substituted prescription drug and type of cannabis used. The distribution of the qualitative responses for brands of prescription drugs was quantified in Nvivo 12.

### Data management

The 52 conditions that the CaM respondents could choose from, when reporting conditions treated with CaM, were categorized as either somatic conditions (*n* = 36) or psychiatric conditions (*n* = 13), except for “chronic pain”, “sleep disturbances”, and “stress”, which were kept as independent categories (See “Appendix 2”). In order to explore CBD-oil use only, we coded a dummy variable for use of CBD-oil only (1) and use of other cannabis products (0). Of the 4.570 respondents who opened the survey, 3.140 answered all questions. Of these, 234 were excluded: 59 were under the age of 18, 115 answered on behalf of someone next of kin, seven had inconsistencies in answers, and 53 were identified as duplicates. Respondents with missing responses to the question on substitution as a motive for use of CaM (*n* = 65) were also excluded, leaving a final sample of 2.841.

The specific brand of prescription drugs entered in the open-ended questions was grouped together under their shared active ingredient. Non-specific entries (e.g., “strong pain killers” or “migraine medication”) were discarded. All entries for drugs that are also available over the counter (e.g., Ibuprofen or Panodil) were assumed to be obtained via prescription, given the nature of the survey questions. A different approach was taken in the “Other” category, where all entries were coded into new categories. Specific entries such as “melatonin” and “imovane” were coded as “sleep medication” together with non-specific entries, such as “sleeping pills”. The most prevalent new categories (*n* > 20) are reported.

### Ethics

Respondents consented to participate and could drop out of the survey at any time before completion. Respondents were not compensated for participating. The data used for this study were collected and stored for monitoring on secure servers, and procedures for data handling and storage were approved by the Danish Data Protection Agency. No ethics evaluation was needed under Danish law.

## Results

The majority of respondents were female (63.2%), and aged 45 years or older (64.1%) (see Table 1). The most prevalent level of education was “medium cycle higher-education” (28.7%) and “vocational secondary education” (18%). Employment status was mixed with 25.9% in full-time employment, 21.8% on disability pension, and 18.4% in reduced employment. All five regions of Denmark were represented in the study.

**Table 1:** Demographics

	Total sample (*n* = 2.841)	Substitution users (*n* = 1.546)	Odds of substituting
	*N* (%)	*N* (%)	OR (95% CI)
**Gender**			
Male	1.020 (35.9)	511 (33.1)	1.00 (reference)
Female	1.769 (63.2)	1.024 (66.2)	1.25* (1.05–1.47)
Other	6 (0.2)	3 (0.2)	1.24 (0.20–7.95)
Missing	19 (0.7)	8 (0.5)	
**Age**			
18–24	126 (4.4)	57 (3.7)	1.00 (reference)
25–34	338 (11.9)	178 (11.5)	1.16 (0.76–1.79)
35–44	555 (19.6)	313 (20.3)	1.20 (0.78–1.84)
45–54	795 (28)	472 (30.5)	1.20 (0.78–1.85)
55–64	681 (24)	365 (23.6)	0.86 (0.55–1.34)
65–74	296 (10.4)	140 (9.1)	0.77 (0.43–1.39)
> 75	47 (1.7)	21 (1.4)	0.71 (0.32–1.57)
Missing	3 (0.1)		
**Current employment**			
Full-time employment	737 (25.9)	340 (22)	1.00 (reference)
Part-time employment	112 (3.9)	61 (4)	1.40 (0.93–2.10)
Student	128 (4.5)	63 (4.1)	1.25 (0.82–1.90)
Unemployed	50 (1.8)	31 (2)	1.93* (1.06–3.53)
Retired (pension and early retirement)	377 (13.3)	180 (11.6)	1.44 (0.95–2.18)
Stay-at-home	23 (0.8)	10 (0.7)	0.94 (0.40–2.19)
Disability pension	620 (21.8)	401 (25.9)	2.21*** (1.75–2.80)
On sick leave	87 (3.1)	43 (2.8)	1.11 (0.71–1.74)
Reduced employment (due to reduced working capacity)	524 (18.4)	323 (20.9)	1.76*** (1.39–2.22)
Other	147 (5.2)	74 (4.8)	1.21 (0.84–3.54)
Missing	36 (1.3)	20 (1.3)	
**Education**			
None	35 (1.2)	17 (1.1)	1.00 (reference)
9th grade	322 (11.3)	165 (10.7)	1.15 (0.56–2.35)
9^th^–-11th (HG, EFG, EGU)	142 (5)	80 (5.2)	1.34 (0.62–2.86)
High school (STX, HHX, HF)	285 (10)	136 (8.8)	1.05 (0.51–2.17)
Vocational secondary education	512 (18)	307 (19.9)	1.68 (0.83–3.40)
Short-cycle higher education	332 (11.7)	181 (11.7)	1.26 (0.61–2.58)
Medium-cycle higher education	814 (28.7)	460 (29.8)	1.48 (0.74–2.98)
Long-cycle higher education	237 (8.3)	119 (7.7)	1.30 (0.62–2.70)
Other	103 (3.6)	53 (3.4)	1.21 (0.55–2.65)
Missing	59 (2.1)	28 (1.8)	
**Region**			
Capital	670 (23.6)	344 (22.3)	1.00 (reference)
Central Jutland	623 (21.9)	329 (21.3)	0.96 (0.77–1.21)
Zealand	558 (19.6)	318 (20.6)	1.14 (0.90–1.44)
Southern Denmark	537 (18.9)	309 (20)	1.19 (0.94–1.51)
Northern Jutland	340 (12)	191 (12.4)	1.16 (0.88–1.51)
Missing	113 (4)	55 (3.6)	

*OR* odds ratio; *CI* confidence interval

*p < 0.05; ***p < 0.001

The most prevalent type of CaM was CBD-oil (65.1%), followed by “hash, pot or skunk” (36.4%), and THC-oil (24.5%), and oil was the most frequent form of intake (56.4%) (see Table 2). Most users had limited recreational experience (63.5%), and the vast majority were current users (91.1%), with no prescription for medical cannabis (91%). A majority of respondents used CaM in the treatment of a somatic condition (73.3%), followed by psychiatric conditions (37.1%), chronic pain (32.8%), sleep disturbances (27.9%), and stress (24.1%) (See Table 2). The most prevalent somatic condition treated with CaM was osteoarthritis (23.2%), and almost equally prevalent among the psychiatric conditions were anxiety (19.6%) and depression (19.5%).

**Table 2:** Patterns of use and illness/es treated

	Total sample (*n* = 2.841)	Substitution users (*n* = 1.546)	Odds of substituting
	*N* (%)	*N* (%)	OR (95% CI)
**Types of cannabis**			
THC-oil	697 (24.5)	415 (26.8)	1.28* (1.07–1.53)
CBD-oil	1.850 (65.1)	1.008 (65.2)	0.96 (0.81–1.13)
Hash, pot or skunk	1.035 (36.4)	566 (36.6)	1.16 (0.97–1.38)
Cannabis based (Marinol, Sativex)	172 (6.1)	105 (6.8)	1.22 (0.89–1.69)
Whole plant trial (Bedrocan, Bediol)	54 (1.9)	34 (2.2)	1.42 (0.81–2.51)
Other	221 (7.8)	124 (8)	1.03 (0.78–1.37)
**Respondents only using CBD oil**	1.086 (38.2)	560 (36.2)	0.79* (0.67–0.93)
**Frequency of use**			
6–7 days a week	2.141 (75.4)	1.197 (77.4)	1.00 (reference)
3–5 days a week	366 (12.9)	187 (12.1)	0.86 (0.69–1.08)
1–2 days a week	162 (5.7)	76 (4.9)	0.78 (0.56–1.08)
A few times a month	92 (3.2)	45 (2.9)	0.83 (0.54–1.27)
Very rarely	50 (1.8)	24 (1.6)	0.74 (0.42–1.31)
Missing	30 (1.1)	17 (1.1)	
**Most frequent form of intake**			
Smoked	686 (24.2)	355 (23)	0.94 (0.76–1.13)
Vaporized	102 (3.6)	54 (3.5)	1.03 (0.69–1.56)
Oil	1.601 (56.4)	861 (55.7)	0.87 (0.74–1.02)
Edibles, suppositories, tea, topical	142 (5)	87 (5.6)	1.37 (0.96–1.95)
Capsules	119 (4.2)	71 (4.6)	1.23 (0.84–1.79)
Other	172 (6.1)	107 (6.9)	1.35 (0.97–1.86)
Missing	19 (0.6)	11 (0.7)	
**Level of recreational experience**			
Novice (lifetime use < 5 times)	1.803 (63.5)	991 (64.1)	1.00 (reference)
Experienced (lifetime use > 5 times)	981 (34.5)	531 (34.4)	1.08 (0.90–1.28)
Missing	57 (2)	24 (1.6)	
**Prescription for medical cannabis**			
No	2.586 (91)	1.381 (89.3)	1.00 (reference)
Yes	238 (8.4)	162 (10.5)	1.68*** (1.26–2.24)
Missing	17 (0.6)	3 (0.2)	
**User status**			
Current user of CaM	2.588 (91.1)	1.414 (91.5)	1.00 (reference)
Former user of CaM	253 (8.9)	132 (8.5)	0.94 (0.72–1.23)
**Illnesses treated with CaM**			
Somatic condition	2.083 (73.3)	1.196 (77.4)	1.44*** (1.21–1.71)
Psychiatric condition	1.055 (37.1)	593 (38.4)	1.15 (0.98–1.35)
Chronic pain	932 (32.8)	642 (41.5)	2.30*** (1.94–2.72)
Sleep disturbances	793 (27.9)	443 (28.7)	1.09 (0.92–1.29)
Stress	684 (24.1)	351 (22.7)	0.91 (0.76–2.94)
**Mean number of conditions treated with CaM**	3.2 (SD 2.5)	3.6 (SD 2.6)***	*z* = − 9.01, *r* = − 0.17

For overview of Somatic and Psychiatric conditions see “Appendix 2”

*OR* odds ratio; *CI* confidence interval

*p < 0.05; ***p < 0.001

### CaM used as a substitute for prescription drugs, and substitution user characteristics

The majority (70.1%) indicated that they had experience using prescription drugs in treatment of their condition(s), of which 77.7% (*n* = 1.546) had used CaM with the explicit purpose of replacing a prescribed drug. Thus, substitution users made up more than half of the total sample (54.6%).

The odds of using CaM as a substitute for prescription drugs were 1.25 times greater among women (95% confidence interval (CI): 1.05–1.47, *p* < 0.05), 2.21 times higher among users on disability pension (95% CI: 1.75–2.80, *p* < 0.001), and 1.76 times higher among users in reduced employment (95% CI 1.39–2.22, *p* < 0.001), compared to non-substitution users. Users with access to medical cannabis via prescription were 1.68 times more likely to be substitution users (95% CI 1.26–2.24, *p* < 0.001), while level of recreational experience was not significantly associated with substitution use (see Table 2). There were no significant associations between using cannabis as substitution and age, education, and region (see Table 1).

Substitution users reported a higher mean of conditions treated with CaM (M 3.6, SD 2.5) compared to other users of CaM (M 3.2, SD 2.5). Substitution users were 1.44 times more likely to use CaM to treat a somatic condition (95% CI 1.21–1.71, *p* < 0.001), and 2.30 times more likely to use CaM to treat chronic pain (95% CI 1.94–2.72, *p* < 0.001) compared to non-substitution users (see Table 2). There were no associations between a substitution motive and the use of CaM to treat “sleep disturbances”, “stress”, or “psychiatric conditions”.

### Prescription drugs substituted and impact of using CaM as a substitute

**Fig. 1:**
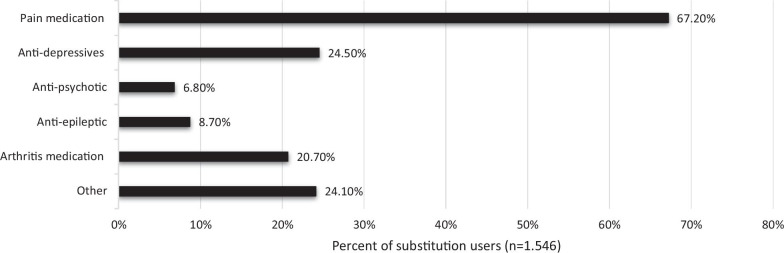
Type of prescription drug(s) substituted with CaM

Pain medication (67.2%) was the most common type of prescription drug substituted, followed by anti-depressants (24.5%), “other” (24.1%), and arthritis medication (20.7%) (see Fig. 1). Specific brands or classes of prescription drugs were listed 1.887 times, of which 1.246 (66%) were pain medication with Tramadol (27.2%) and Morphine (15.5%) as the most common (see Table 3). In the “Other” category, 432 drugs were listed, and the most prevalent medications were “sleep medication” (16%), “ADHD medication” (13.7%), and “anxiety medication” (8.1%).

**Table 3:** Five most prevalent classes of prescription drugs substituted with CaM (brands in parentheses)

	*n* (%)
**Pain medication (*n*** **= 1246)**	1.246
Tramadol (Dolol, Gemadol, Nobligan, Mandolgin, Tadol, Tradolan)	339 (27.2)
Morphin (Contalgin, Depolan, Doltard, Malfin)	193 (15.5)
Paracetamol (Kodipar, Fortamol, Doleron, Pamol, Panodil, Pinex)	175 (14.2)
Ibuprofen (Ipren, Ibumetin, Brufen)	151 (12.3)
Oxycodon (Oxiconti, Oxynorm)	67 (5.4)
**Antidepressants (*n*** **= 209)**	209
Sertralin (Sertralin, Zoloft)	35 (16.8)
Citalopram (Citalopram, Cipramil)	29 (13.9)
Venlafaxin (Venlafaxin, Efexor)	24 (11.5)
Duloxetin (Duloxetin, Cymbalta)	21 (10)
Amitriptylin (Amitriptylin, Saroten)	12 (5.7)
**Anti-psychotic (*n*** **= 82)**	82
Quetiapin (Quetiapin, Seroquel)	32 (39.1)
Chlorprothixen (Truxal)	14 (17.1)
Aripiprazol (Abilify)	5 (6.1)
Mathylphenidat (Ritalin, Concerta)	5 (6.1)
Olanzapin (Olanzapin, Zyprexa)	4 (4.9)
**Anit-epileptic (*n*** **= 114)**	114
Gabapentin (Gabapentin)	44 (38.9)
Pregabalin (Lyrica)	37 (32.7)
Lamotrigin (Lamotrigine)	5 (4.4)
Valproat (Deprakine)	4 (3.5)
Clonazepam (Rivotril)	3 (2.7)
**Arthritis medication (*n*** **= 236)**	236
Ibuprofen (Ipren, Ibumetin, Brufen)	123 (52.1)
Diclofenac (Arthrotec, Diclon, Diclofenac, Voltaren)	16 (6,9)
Methrotrexat (Metex, Meteozane)	16 (6.9)
Paracetamol (Pamol, Panodil)	9 (3.9)
Naproxen (Naproxen)	7 (3)
**Drugs listed in “Other” divided into categories: (***n* **= 432)**	432
Sleep medication (Circadin, Melatonin, Halcion, Imozop, Nitrezepam, Zonoct, Stilnoct, Zolpidem, Propavan, Zopiclone)	69 (16)
ADHD medication (Ritalin, Stratea, Elvanse, Concerta, Medikinet, Motiron Methylphenidate)	59 (13.7)
Anxiety medication (Alprazolam, Alprox, Diazepam, Oxapax, Oxazepam, Lorazepam, Stesolid, Hydroxyzine, Atarax)	35 (8.1)
Pain medication (Panodil, Morphin, Tramadol, Dolol, Ibuprofen)	35 (8.1)
Muscle relaxants (Baklofen, Sirdalud, Klorozoxazon, tizanidin)	25 (5.8)
Migraine medication (Sumatriptan, Dixarit, Migea, Triptaner, Imigran, Zomig, Relpax)	25 (5.8)
Topical medication (Dermovat, Locoid, Daivobet, Calcipotriol, Elocon, Zovir, Xamiol)	20 (4.6)
Blood pressure medication (Amlodipine, Ancozan, Aprovel, Candesartan, Doxasozin, Verapamil)	18 (4.2)
Prednisolone (Adrenal cortex hormone)	17 (3.9)

**Fig. 2:**
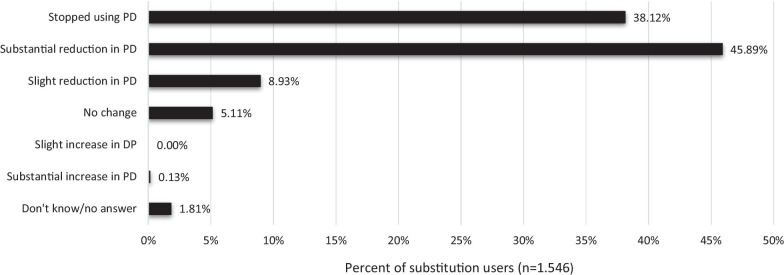
Effect of substitution with CaM on prescription drug (PD) use

Among the 1.546 respondents who reported using CaM as a substitute for prescription drugs, the vast majority had stopped using prescription drugs (38.1%) or reduced their use substantially (45.9%). Very few reported a substantial/slight increase in prescription drug use (0.1%) as a consequence of their use of CaM (see Fig. 2).

### Type of cannabis used as a substitute

CBD-only users made up one third of all substitution users (36.2%). Substitution users were 1.28 times more likely to use THC-oil (95% CI 1.07–1.53, *p* < 0.05) compared to non-substitution users, and less likely to be CBD-only users (OR: 0.79 (95% CI 0.67–0.93, *p* < 0.05). There were no other significant differences in patterns of use between substitution users and non-substitution users (see Table 2).

**Fig. 3:**
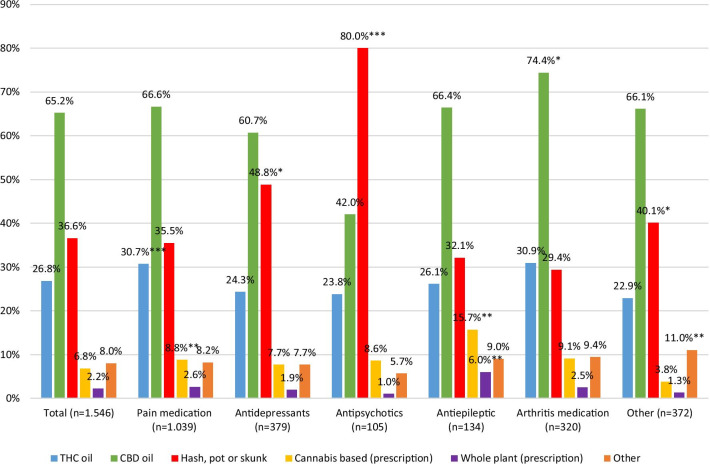
Proportion of types of cannabis used by respondent in each prescription drug category. (*Notes*: *p < 0.05; **p < 0.01; ***p < 0.001. Covariates controlled for: age, gender, and current employment. For odds ratios and confidence intervals, see “Appendix 1”)

Type of cannabis used varied depending on the types of prescription drugs substituted (see Fig. 3). CBD-oil was the most prevalent type of cannabis used among those who substituted arthritis medication (74.4%), pain medication (66.6%), anti-epileptics (66.4%), and anti-depressants (60.7%), but not among those who substituted anti-psychotics (42%), where “hash, pot or skunk” were the most common types used (80%). The odds of using “hash, pot or skunk” were 1.45 times higher among users who substituted anti-depressants (95% CI 1.08–1.95, *p* < 0.05), and 3.37 times higher among users who substituted anti-psychotics (95% CI 1.87–6.08, *p* < 0.001). Users who substituted pain medication were 1.72 times more likely to use THC-oil (95% CI 1.30–2.27, *p* < 0.001), and users who substituted arthritis medication were 1.46 times more likely to use CBD-oil (95% CI 1.08–1.98, *p* < 0.05).

### Experienced effects and side effects

**Fig. 4:**
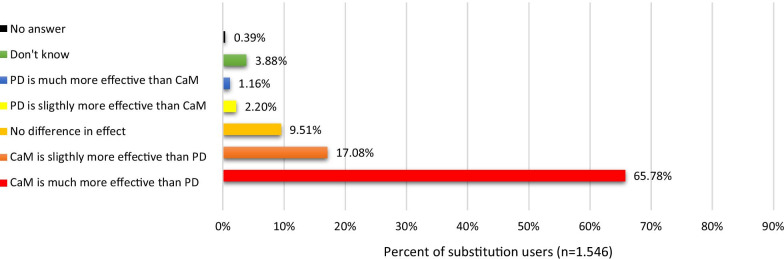
Effects of CaM compared to effects of prescription drugs (PD)

More than half (65.8%) indicated that CaM was a ‘much more effective’ treatment of their condition(s) compared to prescription drugs. A minority indicated that prescription drugs were ‘slightly more effective’ (2.2%) or ‘much more effective’ (1.2%) compared to CaM (see Fig. 4).

**Fig. 5:**
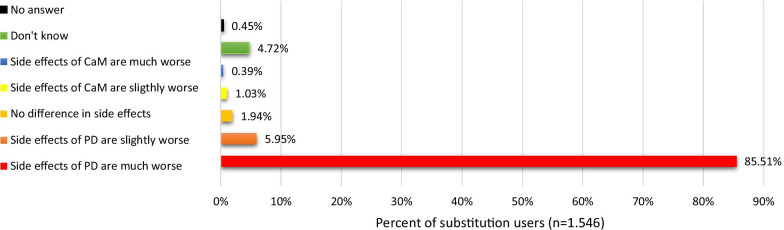
Side effects of CaM compared to side effects of prescription drugs (PD)

When comparing side effects between prescription drugs and CaM, the majority (85.5%) indicated that the side effects of prescription drugs were ‘much worse’ than the side effects of CaM, and a minority reported that the side effects of CaM were ‘slightly worse’ (1%) or ‘much worse’ (0.4%) than the side effects of prescription drugs (see Fig. 5).

## Discussion

To our knowledge, the present study is the largest study to date on self-reported use of CaM as substitution for prescription drugs in a European sample. Further, the study is the first to explore the type of cannabis used as a substitute across different types of prescription drugs, providing valuable insights into user perspectives on CaM as a substitute for prescription drugs in treating somatic and mental health conditions.

Our findings show that substitution of prescription drugs is a leading motive among users of CaM in a Danish convenience sample, and that this practice is more common among women, people with reduced working capacity due to illness, and people with chronic pain and other somatic conditions. We found that pain medication by far was the most common type of prescription drug substituted, followed by anti-depressants and arthritis medication, and that Tramadol was the most common class of pain medication that was substituted with CaM. Furthermore, the self-reported substitution effect was considerable, as the vast majority of substitution users reported either a substantial decrease in, or cessation of, prescription drug use as a consequence of their use of CaM. Across prescription drug categories, we found that CBD-oil was the most common form of cannabis used as substitution, except for people who substituted anti-psychotic medicines, where the use of ‘hash, pot or skunk’ was the most prevalent. A majority of the substitution users reported that CaM was much more effective in treating their conditions compared to the prescription drugs that they had used, and a large majority reported that the side effects from their use of prescription drugs were much worse compared to the side effects when using CaM.

### Prevalence of substitution and characteristics of substitution users

Studies from Canada and the USA indicate that use of CaM as a substitute for prescription drugs is prevalent among users with legal access to cannabis [24, 26–28, 30, 64, 84, 85]. Our study adds to this literature by showing that substitution of prescription drugs is also a prevalent motive among users of CaM in a European sample, where the majority of substitution use takes place outside a legal medical setting. While a recent international cross-sectional survey found that substitution of prescription drugs was not significantly related to having legal access to medical cannabis [29], we found that users with legal access to CaM were significantly more likely to be substitution users compared to users without legal access. This difference may be partly explained by the fact that in Denmark, legal medical cannabis is only recommended for specific conditions when other treatment options have proven inadequate [65]. Our finding that users with limited recreational experience with cannabis are just as likely to use cannabis as a substitute for prescription drugs as experienced recreational cannabis users is in line with previous research on substitution as a motive among prescription CaM users [26]. Overall, the present study adds to the growing body of research indicating that use of cannabis as a substitute for prescription drugs is a prevalent phenomenon.

Our finding that women are more likely than men to substitute prescription drugs with CaM resembles previous studies [24, 28, 29] and indicates that there may be a more general pattern of gender differences related to the use of CaM. Of note, a cross-sectional survey of cannabis users from the USA found that female users report significantly lower frequency and quantity of cannabis use and significantly higher rate of medicinal use for anxiety, irritable bowel syndrome, nausea, anorexia, and migraines compared to men [86]. Further, a qualitative study on legal medical cannabis use, also from the USA, found that gender may influence patterns and practices of use, as the narrative of female users included more collaboration with health care providers compared to the narrative of male users [87]. While other studies have found that older CaM users are more likely to be substitution users [29], our study showed that a substitution motive of CaM was not significantly related to age. The fact that substitution users in our study were more likely to be on disability pension or in reduced employment may indicate that these people encounter treatment barriers in the healthcare system that ‘push’ them towards alternative types of treatment. One such barrier could be the cost of treatment, as the current instability in the prescription drug market with large fluctuations in price [88, 89] may further an economic substitution motive among some low-income patients. However, the use of unregulated cannabis may still be more expensive for most compared to the price of prescription drugs, as government reimbursement of medicine costs in Denmark are substantial [90]. Furthermore, we found that CaM users who treat chronic pain were more likely to be substitution users, which may indicate insufficient treatment options for this patient group, as those suffering from non-malignant chronic pain are potentially undertreated [91, 92].

### Type of prescription drug(s) substituted and the impact of substitution

Our finding that pain medications, particularly opioids, were the most common prescription drugs substituted with CaM, followed by anti-depressives and arthritis medication, corresponds with our findings on chronic pain, arthritis, and depression as the most frequent motives for use of CaM. These findings reflect findings from other survey studies; in a large international cross-sectional survey on cannabis users, Corroon et al. [29] found that opioids (13.6%), anxiolytics (12.7%), and antidepressants (12.7%) were the most common classes of prescription drugs substituted with cannabis, and a Canadian cross-sectional survey of medical cannabis patients found that opioid medications accounted for 35.3% of all prescription drug substitutions, followed by antidepressants (21.5%) [24].

The fact that Tramadol was the most common type of prescription drug substituted in our study may in part be explained by the prevalent use of Tramadol in treatment of non-malignant pain in Denmark [70]. However, it may also be related to the decision by the Danish Medicines Agency to surveil prescription patterns of Tramadol among physicians in September 2017, following a rise in Tramadol use [70] and growing concern among clinicians’ regarding the abuse potential of Tramadol [93]. It is likely that this decision reduced access to prescribed Tramadol for some patients, who subsequently turned to CaM in order to treat their pain condition. In fact, the number of Tramadol users in Denmark decreased for the first time since 2008 in 2017 [94], and continued to decrease from 2017 to 2018 (23% reduction in Tramadol users) [95]. Indeed, problematizing and reducing opioid prescriptions leaves a vacuum that may motivate some patients to seek other therapies such as CaM [96]. Interestingly, the increased use of Tramadol in Denmark earlier occurred as a consequence of another vacuum in the management of non-malignant pain caused by the problematization of nonsteroidal anti-inflammatory drugs (NSAIDs) and the discovery of serious long-term side effects of these drugs [70]. Thus, there seems to be a “cycle of vacuums” in the treatment of non-malignant pain, underscoring the need to rethink the management of non-malignant pain in the Danish health care system [92], as this patient group is potentially undertreated [91].

Our findings on the cessation of and substantial decrease in prescription drug use show a considerable reported substitution effect related to the use of CaM, which is echoed in other studies on the reported substitution effect of CaM among patients with access to medical cannabis. In a survey of dispensary members in New England, the majority reported a decreased use of opioids (76.7%), anxiety medication (71.8%), migraine medication (66.7%) and sleep medication (65.2%) [26]. Similarly, in a survey of American medical cannabis users with chronic pain, the majority of users reported complete cessation of opioids (72%) benzodiazepines (68%), NSAIDs (44%), gabapentanoids (74%), disease-modifying antirheumatic drugs (80%), Serotonin–Norepinephrine Reuptake Inhibitors (78%), and Selective Serotonin Reuptake Inhibitors (80%) [28]. The link to a decrease in opioid use has also been shown over time in a small cohort study in New Mexico, comparing 37 chronic pain patients enrolled in a medical cannabis program to 29 non-enrolled chronic pain patients over the course of 21 months [35]. Findings from this study showed clinically and statistically significant associations between medical cannabis enrollment and opioid prescription cessation and reduction, as well as improved quality of life. Thus, findings from our study add to the growing body of research indicating that from a user perspective, CaM has a substantial substitution effect for a variety of prescription drugs, most notably opioids.

### Type of cannabis used as a substitute for prescription drugs

The findings that, in our sample, CBD-oil is the most prevalent type of cannabis used as a substitute for prescription drugs, and that one third of the substitution users used CBD-oil only, are in accordance with findings from a recent survey of 1.483 medicinal CBD users in the USA where CBD was used as a specific therapy for medical conditions, particularly pain and inflammatory disorders, as well as anxiety, depression, and sleep disorders [97]. In the same study, the majority (65.3%) reported that CBD treated their condition(s) moderately or very well without the use of conventional medicine, and 30.4% reported that CBD was effective in combination with conventional medicine. Our findings are also in accordance with a recent Italian study, where the unintended legalization of CBD-based cannabis products with less than 0.6% THC was associated with a significant decrease in the sale of prescription drugs, particularly of anxiolytics, sedatives, and anti-psychotics [40]. This is of particular public health interests, as CBD has been shown to have a better safety profile in terms of side effects and abuse potential, relative to THC [42, 98, 99]. In line with this, a recent review and meta-analysis of clinical trials found that CBD was well tolerated and had few serious side effects across medical conditions [100]. The safety profile of CBD-based cannabis products may also be superior in terms of toxicology and abuse potential compared to some of the prescription medication that is substituted, such as opioids and benzodiazepines [49, 52, 53].

From a public health perspective, the problematic aspects of medicinal use of low THC/high CBD cannabis products are also, and maybe more, related to the fact that these products are unregulated and used without medical supervision [101]. Indeed, use of unregulated cannabis products increases the risk of consuming hazardous contaminants, such as fungi, bacteria, pesticides or heavy metals [1, 102] or consuming a product with undesired psychoactive effects. Interestingly, a recent examination of CBD-oils available in Denmark by Department of Forensic Medicine in Odense, revealed that 38% of CBD-oils tested contained between 0.2% THC and 1.2% THC, despite being advertised as below 0.2% THC^1^ [103]. Thus, users of CBD-oil may unknowingly use products that are illegal to consume.

Our finding that users of CaM, who substituted anti-depressants or anti-psychotics, are significantly more inclined to use “hash, pot or skunk” compared to other substitution users is of particular interest to public health, as the THC content in skunk is high, and the THC content in cannabis resin (hash) and herbal cannabis has increased markedly in the last decades in Europe [104] and the USA [105, 106]. In Denmark, we found a threefold increase in THC concentration in seized hash from 2000 (mean: 8.3%) to 2017 (mean: 25.3%), while CBD levels remained stable (mean around 6%) [107]. This trend is concerning, as increasing evidence suggests that exposure to high-THC and low-CBD cannabis products is associated with higher risk of cannabis-related harms, such as cannabis dependence [108–110], psychosis [111, 112], and cognitive impairment [99, 113], compared to low-THC and high-CBD products. The magnitude of the problem is further underlined by findings from naturalistic studies on users of smoked cannabis products, which indicate that users do not fully adjust their use to differences in THC concentration, suggesting that users of more potent products are exposed to higher levels of THC [114, 115]. Thus, it is possible that use of high THC cannabis products as a substitute for prescription drugs may exacerbate the condition that is the target of the treatment, particularly in relation to treatment of psychotic disorders.

In sum, there is considerable complexity related to the use of cannabis as a substitute for prescription drugs, as an evaluation of whether cannabis is in fact “the lesser of two evils” depends on various factors, such as the cannabinoid composition in the type of cannabis used, the dosage, and the type of prescription drug that is substituted. Recently, another “lesser of two evils”-dynamic is emerging from cannabinoid research, as it is increasingly plausible that low-THC cannabis products are less harmful compared to high-THC cannabis products, and that increased availability of low-THC cannabis products may hold a potential for harm reduction among users of high-THC cannabis products [42]. In line with this reasoning, the unintended legalization of low THC-cannabis products in Italy, was not only associated with a significant decrease in prescription drug use, but also with a decrease in confiscations of illegal cannabis and drug-related arrests [116], indicating a substitution effect of introducing low THC-cannabis products on the consumption of conventional illegal cannabis products.

### Experienced effects and side effects

Findings from our sample show that most substitution users find CaM more effective in managing their condition(s) compared to prescription drugs, and that an overwhelming majority found CaM to have a better side effect profile compared to the prescription drugs that they had been prescribed for their condition(s). This is in line with recent findings from Canada and the USA. In a cross-sectional survey, Canadian medical cannabis users listed, ‘relative safety of cannabis to prescription drug’, ‘fewer adverse side effects’ and ‘better symptom management’ as their top three reasons for using CaM as a substitute for prescription drugs [24]. In a survey of medical cannabis patients in California who used cannabis as a substitute/in conjunction with opioid-based pain medication, 80% found that cannabis was more effective than opioids for pain, and 92% that the side effects of cannabis were more tolerable than opioids [27]. Moreover, in a survey of American medical cannabis users with chronic pain, respondents listed ‘fewer side effects’ and ‘better symptom management’ as their top reasons for using medical cannabis as a substitute for prescription drugs [28]. Lastly, a qualitative study on cannabis users in San Francisco showed similar perceptions among substitution users who found cannabis to be a safer and more effective alternative compared to prescription drugs [31]. It is interesting that substitution users in our and several other studies rate the side effect profile of CaM higher than its effects, when comparing cannabis to prescription drugs. This suggests that substitution users may have the same “ lesser of two evils”-perspective on the medical utility of cannabis that was documented among some physicians in Israel [57], where cannabis becomes medicine not only on the basis of what it *is* in terms of effects, but on what it *is not* in terms of side effects when compared to prescription drugs. Considering the growth in use of CaM, it is likely that this perspective will result in an increasing number of people seeking information and advice about the effectiveness of CaM and use CaM as a substitute for prescription drugs, even in the absence of rigorous clinical trials and despite lack of legal access to medical cannabis. Future research is needed to assess effects and side effects of long-term use of CaM from longitudinal studies. Furthermore, placebo-controlled clinical efficacy trials are needed to explore the effects of cannabis beyond placebo, and current barriers to whole plant cannabis research need to be addressed [1].

### Limitations

Our study has several limitations. First, this self-selected convenience sample is limited by selection bias, as it likely weighs towards successful users of CaM, users with internet access, a familiarity with online surveys, users engaged with the topic on social media, and with the resources necessary to answer such surveys. Therefore, the survey may not be representative of the population of CaM users or substitution users [117]. Second, the data used in the study may be subject to self-reporting biases, such as recall bias, confirmation bias, placebo effects or social desirability bias [118]. For example, users are likely to have optimistic expectations regarding the efficacy of CaM [119] and may exaggerate positive effects of CaM or under-report adverse effects. Also, recall bias may be more salient for the small group of respondents who were not current users. Third, although duplicates were excluded from the analyses, we cannot rule out multiple responses from the same person, as IP-addresses were not accessible to researchers. Fourth, the cross-sectional study design lacks a temporal dimension, and we do not know if the reported cessation or reduction in prescription drug use is sustained over time. Fifth, findings on prescription drugs substituted may be skewed by the fact that “sleep medication”, “ADHD medication” and “anxiety medication” were not presented to respondents as independent categories. Sixth, the experienced effects and side effects of CaM and prescription drugs could potentially be affected by pharmacokinetic interactions between cannabis products and prescription drugs when taken simultaneously [120, 121]. Also, the reported effects and side effects were a mapping of substitution users experiences with various types of prescription drugs and different subtypes of cannabis, which likely vary in terms of effect and side effect profile.

## Conclusions

The use of CaM as a substitute for prescription drugs is a leading motive among Danish users of CaM in our sample. Pain medication was the most prevalent prescription drug substituted with CaM, followed by antidepressants and arthritis medication. Tramadol was the most common pain medication substituted with CaM, which may be related to a change in prescription practices for Tramadol in Denmark. Across prescription drug categories, CBD-oil was the most prevalent type of cannabis used as substitution, except for anti-psychotics, where “hash, pot or skunk” were the most common types used, which is concerning due to the high levels of THC, particularly in hash and skunk. Substitution users reported substantial decrease or cessation of prescription drug use, and a greater effect and far better side effect profile of CaM compared to prescription drugs. Thus, from the perspective of substitution users, CaM may be viewed as the lesser of two evils compared to prescription drugs. More research is needed on the long-term consequences of use of CaM, including the impact from low and high THC cannabis products on specific somatic and mental health conditions.

## Data Availability

The datasets generated and/or analyzed during the current study are not publicly available as it is part of an ongoing PhD project.

## Appendix 1: Odds of substituting the five classes of drugs related to recreational experience and type of cannabis used, controlled for age, gender and employment

**Table:**

	Pain medication (*n* = 1.039)	Anti-depressive (*n* = 379)	Anti-psychotic (*n* = 105)	Anti-epileptic (*n* = 134)	Arthritis (*n* = 320)	Other (*n* = 372)
Type of cannabis used						
THC-oil	**1.72*** (1.30–2.27)**	0.91 (0.68–1.22)	0.83 (0.49–1.41)	0.90 (0.59–1.37)	1.19 (0.89–1.57)	0.92 (0.69–1.24)
CBD-oil	1.08 (0.84–1.40)	1.04 (0.79–1.37)	0.75 (0.47–1.20)	1.03 (0.69–1.55)	**1.46* (1.08–1.98)**	1.11 (0.84–1.48)
Hash, pot or skunk	1.23 (0.92–1.64)	**1.45* (1.08–1.95)**	**3.37*** (1.87–6.08)**	0.67 (0.42–1.06)	0.90 (0.66–1.25)	**1.43* (1.06–1.95)**
Cannabis based medicines	**2.68** (1.48–4.88**)	1.18 (0.72–1.94)	0.88 (0.37.2.09)	**2.59** (1.49–4.51)**	1.21 (0.76–1.95)	0.58 (0.31–1.08)
Whole plant	1.65 (0.68–4.01)	0.79 (0.32–1.92)	0.32 (0.04–2.58)	**3.28** (1.39–7.73)**	1.02 (0.44–2.37)	0.59 (0.22–1.62)
Other	1.17 (0.76–1.81)	1.06 (0.67–1.69)	0.68 (0.27–1.71)	1.11 (0.58–2.12)	1.29 (0.82–2.03)	**1.80** (1.17–2.76)**

**p* < 0.05; ***p* < 0.01; ****p* < 0.001

## Appendix 2: All five categories

Somatic condition (36): Acne, Aids, Alzheimer’s, Asthma, Chrons disorder, Herniated disc, Dystonia, Eczema, Epilepsy, Endometriosis, Fibromyalgia, Osteoarthritis, Rheumatoid arthritis, Psoriatic arthritis, Cataracts, Hepatitis, Brain damage, COPD, Cancer, FE(chronic fatigue), Chronic nerve inflammation, migraine, Menstrual pain, Multiple sclerosis, Neurodermatitis, IBS, Lupus (SLE), Palliation, Parkinson’s, peripheral neuropathy, Psoriasis, Spinal cord injury, pain following operation, pain following an accident, tinnitus, trigeminal neuralgia.

Psychiatric condition (13): ADHD, Anxiety, dependence (alcohol), dependence (hard drugs), dependence (prescription drugs), Anorexia, Autism, Bipolar disorder, Depression, OCD, PTSD, Schizophrenia, Tourettes/tics.

Potential comorbidities (3); Chronic pain, Sleep disturbances, Stress.

## Footnotes

1 cannabis with less than 0.2% THC is legal to possess.
